# Opportunities and challenges for countermeasures against Rift Valley fever virus: A quintessential One Health pathogen

**DOI:** 10.1371/journal.pntd.0014645

**Published:** 2026-08-17

**Authors:** Brian E. Dawes, Keli N. Gerken, Bernard Bett, A. Desiree LaBeaud

**Affiliations:** 1 Division of Infectious Diseases and Geographic Medicine, Department of Medicine, Stanford University School of Medicine, Stanford, California, United States of America; 2 Institute of Infection, Veterinary and Ecological Sciences, University of Liverpool, Liverpool, United Kingdom; 3 International Livestock Research Institute, Nairobi, Kenya; 4 Infectious Disease Epidemiology, Wageningen University and Research, Wageningen, The Netherlands; 5 Division of Pediatric Infectious Diseases, Department of Pediatrics, Stanford University School of Medicine, Stanford, California, United States of America; Institute of Continuing Medical Education of Ioannina, GREECE

## Abstract

Rift Valley fever virus (RVFV) is a zoonotic arbovirus transmitted by a wide range of mosquito vectors present across the African continent. RVFV has resulted in large-scale epidemics in East, West, southern, and northern Africa with additional epidemics affecting the Arabian Peninsula, Mayotte, and Comoros. In some areas, rather than large-scale isolated outbreaks, RVFV causes a fluctuating low-level endemic burden affecting livestock populations by resulting in pregnancy loss and significant production losses. RVFV risk extends into the human population when it is transmitted either by vector-borne transmission or direct contact with infected animals or animal products such as blood, meat, or milk. Following infection, 2%–3% of humans can develop severe disease manifestations including encephalitis/meningitis, hemorrhagic fever, and retinitis, with visual deficits commonly reported among survivors. While RVF cases in animals and humans have been spatially linked, the proportion and risk associated with spillover events is poorly classified. Given RVFV’s dual threat to human and livestock health, it has been designated a priority pathogen by WHO for development of countermeasures. This review aims to summarize current knowledge of RVFV epidemiology, infection, and control as well as highlight key recent advances in the understanding of epidemiology and vaccine development. Multiple next-generation vaccine candidates have advanced to phase two clinical trials which offer hope for approved and commercially available human vaccines in the coming decades. However, despite these promising advancements, key gaps in knowledge remain, particularly regarding the total burden and geographic distributions of endemic and epidemic infections, shifting epidemiologic patterns, optimal vaccine use cases, and the effects of RVFV infection on pregnancy in humans living in hyperendemic regions. Many gaps in understanding persist, and RVFV remains a quintessential One Health pathogen requiring coordinated human, animal, and environmental investigation and collaboration to translate scientific advances into effective national public health strategies.

## History

The historic emergence of Rift Valley fever virus (RVFV) and subsequent spread across the African continent represent the consequences of a highly dynamic pathogen capable of insidious spread across great distances where cases either go un-noticed or result in a large-scale epidemic. RVFV infection was first described during a 1931 epizootic affecting sheep herds in the Lake Naivasha region of Kenya [1]. Affected herds were described as suffering from high juvenile mortality with high rates of abortion among pregnant animals, and the authors presciently noted that this epizootic followed a period of higher-than-average rainfall suspicious for vector-borne transmission. During this epizootic, the first record of human Rift Valley fever (RVF) disease was described among the scientists investigating the outbreak who developed fevers, rigors, arthralgias, headaches, and visual deficits. These authors noted that sheep herders were also affected but provided less clinical detail [1]. In this initial description, the authors experimentally infected a Kenyan patient in a Nairobi hospital suffering from malaria infection based on anecdotal reports that RVFV infection improved malaria outcomes, yet this patient developed severe infection with hemorrhagic manifestations. Serum from this human patient was subsequently shown to induce RVF in sheep fulfilling Koch’s postulates, and the authors noted similarities with sandfly fever, the first attempt at potential classification, which was surprisingly accurate to the genus level. Initial RVFV descriptions, despite colonial prioritization on livestock and European patients and an ethically dubious human challenge study, offered a fairly comprehensive description of many key aspects of RVFV by highlighting the likely arboviral etiology, close association with heavy rainfall, and clinical descriptions of human and animal disease with severe manifestations. Subsequent retrospective analyses uncovered reports from as early as 1912 of a similar epizootic ovine hepatitis in the Rift Valley region of Kenya [2], while genetic analysis suggests that RVFV initially emerged in East or South Africa in the late 1800s to early 1900s, possibly due to colonial agricultural practices introducing large immunologically naïve livestock herds to the region [3,4]. Over the subsequent decades, major RVFV epidemics were noted across Sub-Saharan Africa, with major foci in the Senegal River basin, East Africa (Kenya, Tanzania, and Somalia), and South Africa (South Africa and Zimbabwe) [5].

The first RVFV epidemic outside of Sub-Saharan Africa occurred in Egypt in 1977–1978, and resulted in an estimated 200,000 human cases and 598 deaths with a previously unreported high degree of morbidity and mortality [6,7]. This epidemic provided many clinical descriptions and definitions of severe disease manifestations including meningitis/encephalitis, hemorrhagic fever, and retinitis. Additional large RVFV epidemics affected northern Kenya (Garissa district) and southern Somalia in 1997–1998 with an estimate of 89,000 human cases with 478 reported deaths [8]. East Africa again experienced a large multi-national epidemic in 2006–2007 [9].

RVFV was reported outside of Africa for the first time during a 2000–2001 epidemic in Saudi Arabia and Yemen resulting in nearly 2,000 reported human cases and 245 deaths [10]. The livestock trade from East Africa has been implicated in the initiation of this outbreak based on genetic characterization of viral isolates [11]. Epidemics in African nations in the Indian Ocean (Madagascar, Comoros, Mayotte) were also thought to have originated from East African livestock trade [12–14]. While livestock trade has been consistently linked to RVFV introductions, a single human travel-associated case of RVFV was reported in China highlighting the growing concern for additional RVFV dissemination to new regions [15]. To this end, serologic surveys have found evidence of more widespread RVFV exposure in the Middle East including Türkiye and Iran suggesting potential further dissemination [16,17].

## Description of the pathogen

### Genomic organization, cell entry, replication, and packaging

RVFV is a tri-segmented negative-sense RNA virus in the genus *Phlebovirus*, family *Phenuiviridae*. The RVFV genome is composed of three genomic segments; a 6.4kb large (L) segment, a 3.2kb medium (M) segment, and a 1.7kb small (S) segment which utilizes an ambisense coding strategy [18]. The L segment encodes the viral RNA-dependent RNA polymerase (L protein) [19]. The M segment encodes a single open reading frame with five in-frame start codons resulting in four polyproteins which are cleaved by host-cell proteases. The primary proteins encoded on this segment are the surface glycoproteins Gn and Gc. Accessory proteins produced by alternate start codon usage upstream of Gn/Gc include the 78 KDa protein, the nonstructural protein NSm, and a truncated NSm’ [20]. The ambisense S segment encodes the viral nucleocapsid protein (N) in the negative-sense, while encoding a nonstructural protein (NSs) in the positive-sense [21].

**Fig 1:**
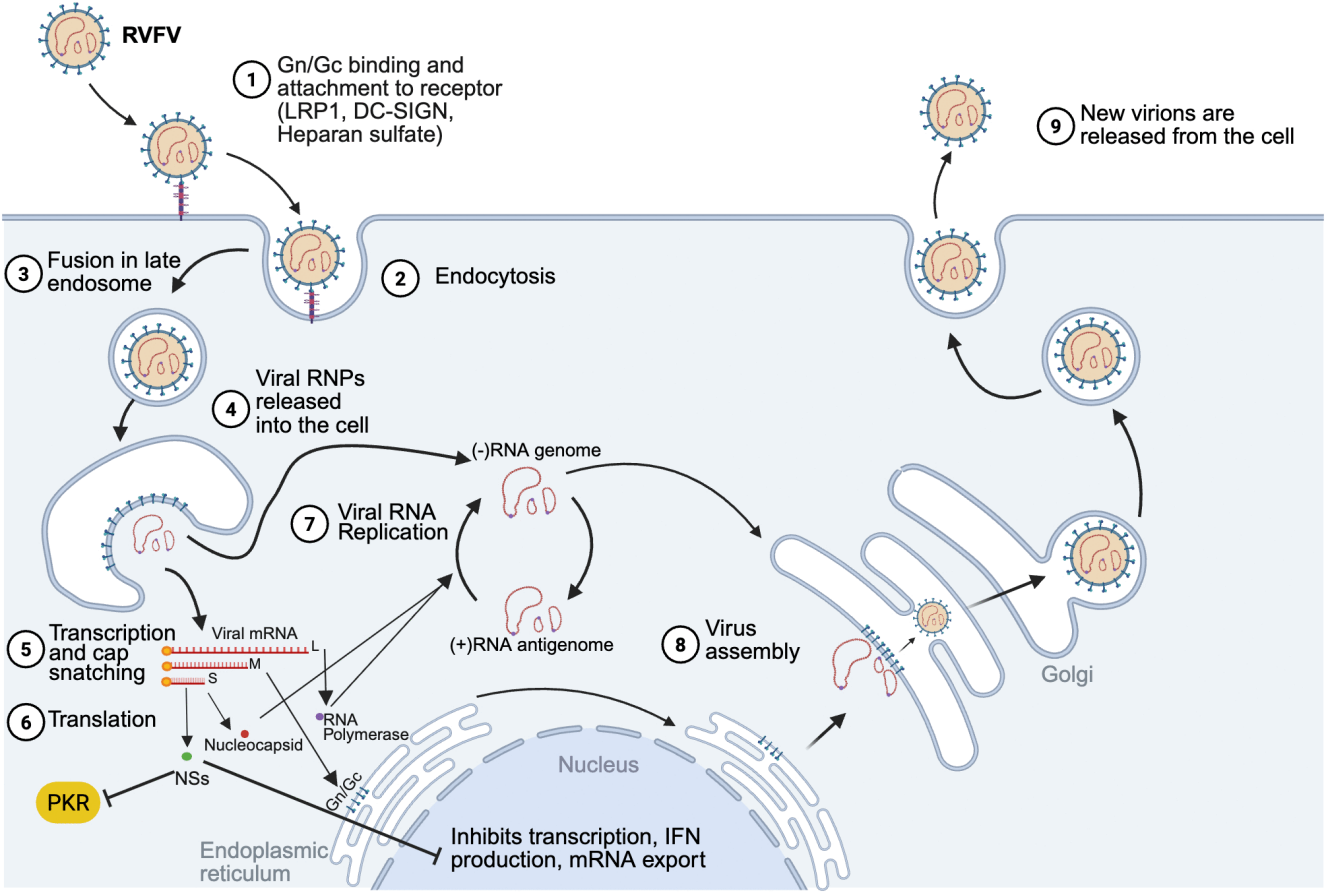
Rift Valley fever virus lifecycle. (1) RVFV glycoproteins (Gn/Gc) attach to host cell receptors including LRP1, DC-SIGN, and heparan sulfates. (2) The virus then enters the host cell via endocytosis which may be clathrin- or caveolin-mediated. (3/4) Upon acidification of late endosomes (pH < 5.7) Gc induces membrane fusion releasing viral genomic RNA into the cytoplasm where it undergoes transcription and replication. (5) Viral transcription produces viral mRNAs with the viral RNA-dependent RNA polymerase facilitating cap snatching from host-cell mRNAs. (6) Viral proteins are produced via translation, with Gn/Gc targeted to the endoplasmic reticulum. Structural N and L proteins participate in genome replication, and NSs serves an interferon antagonist and modulates host-cell gene expression. (7) Viral genome undergoes replication in the cytoplasm along with antigenomic intermediates. (8) Virions undergo assembly in the Golgi and (9) are released from the host cell. Created in BioRender. Dawes, B. (2026) https://BioRender.com/98ep8sl.

Host-cell entry and attachment are facilitated via Gn/Gc complexes. The major RVFV receptor has been identified as low-density lipoprotein-related-receptor (LRP1), with high hepatic expression of this receptor likely responsible for the hepatotropism exhibited by RVFV [22,23]. Additional receptors have been identified including heparan sulfate and dendritic cell-specific intercellular adhesion molecule-3-grabbing non-integrin (DC-SIGN), which may be a major determinant for early macrophage and dendritic cell infection [24]. Following attachment, entry is mediated by endocytosis, and acidification of late endosomes triggers fusion with Gc which functions as a class-II fusion protein [25]. The functions of the non-structural proteins NSs/NSm are largely as the major immune antagonists and virulence factors for RVFV and are discussed later. The 78KDa protein appears largely dispensable for mammalian cell replication, but necessary for efficient mosquito replication; however, its function is still largely unclear [26,27]. The RVFV lifecycle is depicted in Fig 1.

### Viral diversity and reassortments

RVFV exhibits limited genetic diversity and a low evolutionary rate [3,4]. RVFV has been classified into 15 lineages (A–O) found throughout the African continent [28]. Importantly, multiple phylogenetic schemes reveal the co-circulation of lineages in endemic settings, suggesting multiple introductions via animal trade [3,4,28]. Additionally, during epidemics, multiple sub-lineages have been identified highlighting ongoing evolution during interepidemic periods [29]. Lineage C viruses appear to be the most common lineage in Africa, having originated around Zimbabwe in the 1970s, and subsequently becoming particularly associated with East Africa; it also has higher substitution rates suggesting ongoing evolution [4]. Lineage C was also responsible for the Arabian and Mayotte epidemics, and most isolates in Madagascar, supporting the hypothesis that livestock trade from East Africa was the origin of these epidemics. Lineage H is strongly associated with South Africa and has resulted in recent West African epidemics. The notable 1977 Egyptian epidemic was due to lineage A. The clinical significance of varying lineages remains unclear and should be assessed further given that multiple *in vivo* studies in animal models have suggested possible differences in virulence and disease manifestations between field isolates [30].

Reassortments in nature appear rare but have been identified from clinical and field isolates [31]. Notably, reassortments with segments from attenuated veterinary vaccines have been identified in field isolates [28]. The risk of novel reassortments (including of attenuated vaccines) of RVFV to produce unique isolates or even novel viruses remains a potential risk requiring continued surveillance and study.

## Epidemiology

### RVFV transmission and ecology

**Fig 2:**
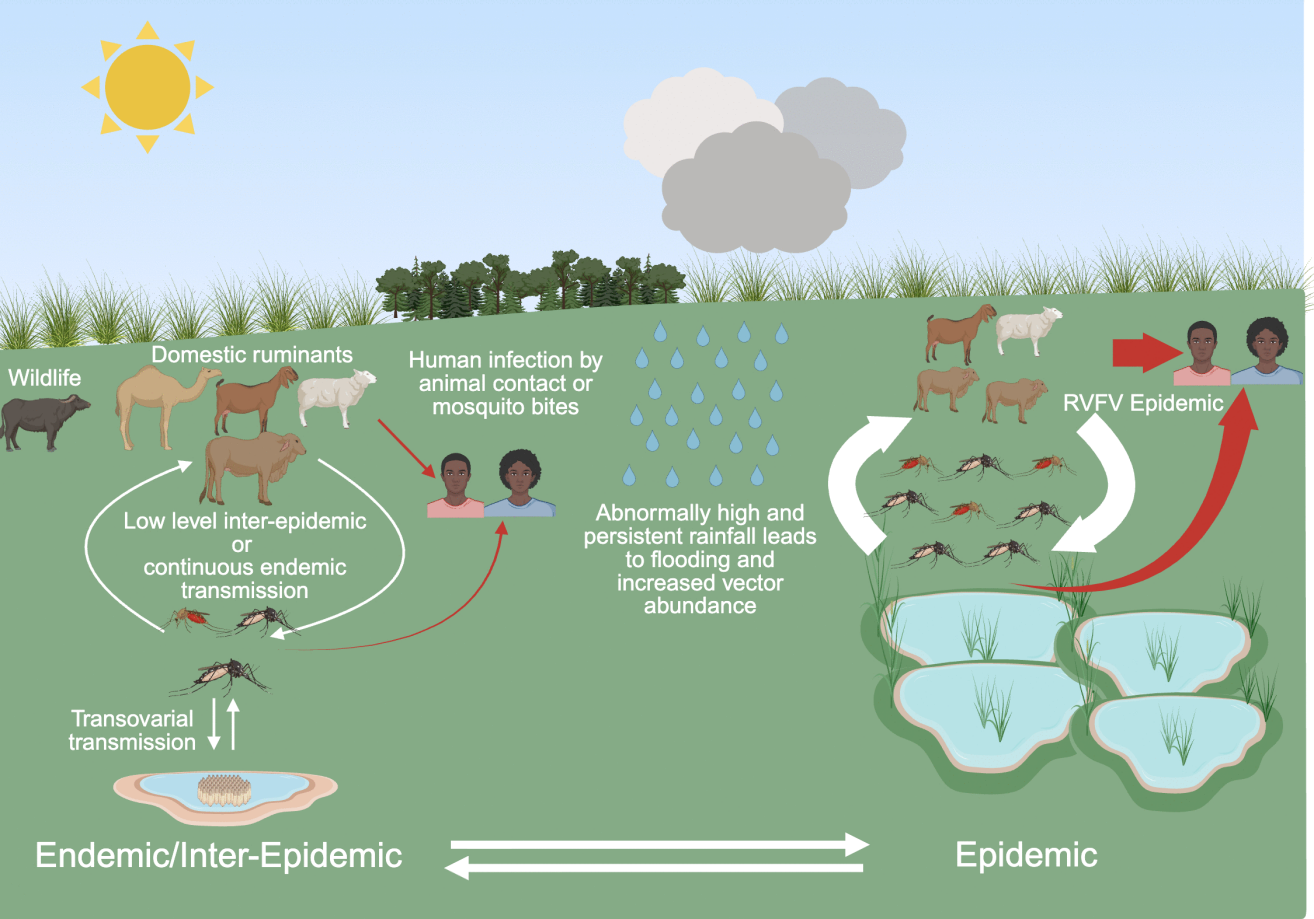
Rift Valley fever virus transmission. RVFV transmission exists on a spectrum between endemic/inter-epidemic transmission and epidemic transmission. Depending on local ecology RVFV may be maintained in mosquito populations via transovarial transmission in floodwater *Aedes* mosquito species and circulate at low levels among livestock and/or wildlife species. In some endemic settings, continuous low-level circulation in animals may be sufficient to maintain transmission independent of transovarial transmission and without evident epidemics. Sporadic cases or small outbreaks of human infections may occur in these settings. In epidemic-prone regions, abnormally high rainfall may result in flooding and increased vector abundance leading to high levels of vector-borne transmission among animals and humans. Humans may be infected via vector-borne transmission or direct contact with infected animals. Created in BioRender. Dawes, B. (2026) https://BioRender.com/0inna05.

RVFV transmission occurs in two major host systems; domestic livestock (cattle, sheep, goats, and camels) and humans. Livestock-to-livestock transmission is thought to occur primarily via vector-borne transmission as immunosuppressed lambs housed with infected animals did not develop infections [32]. It is, however, unknown if livestock can transmit RVFV directly to other livestock through tissues and fluids with high viral titers such as abortion material. On the other hand, human infections are known to be mediated by both vector-borne transmission and direct contact with infected livestock and animal products [33]. RVFV also circulates among a wide variety of wildlife, which may be important for interepidemic maintenance and serve as a risk for livestock infection in regions where wildlife and livestock interact in shared host-vector networks, although the significance of this interaction remains uncertain [34]. A simplified summary of RVFV transmission is provided in Fig 2.

The traditional model of vector-borne emergence of RVFV involves floodwater *Aedes spp.* as the principal vectors capable of transovarial transmission and viral quiescence [35,36]. Here, drought-resistant infected *Aedes* eggs hatch following periods of heavy rainfall and flooding, especially during El Niño events, and this increased vector abundance leads to epizootics/epidemics. This traditional classification also describes secondary vectors such as *Culex spp*. that can amplify transmission (such as in Egypt where *Culex pipiens* appeared to be the primary vector involved in transmission) [37]. However, as interepidemic and hyperendemic transmission becomes increasingly identified, the role of other mosquito species is likely more important than previously realized with regional differences in vector ecology. In hyperendemic settings, year-round mosquito transmission appears likely [38]. Recent proposed models of endemic reservoir systems have suggested systems in which transovarial transmission combined with circulation in livestock and/or wildlife or systems in which mosquito-livestock and/or mosquito-wildlife transmission alone are sufficient for maintenance which vary by region [34]. To date, over 30 species of mosquitoes have been identified as theoretical RVFV vectors [35]. Climate change will likely result in changes to vector-borne transmission patterns with the potential for extended range and altered epidemiologic patterns [39].

Human transmission risk is challenging to dissect as humans can become infected via the bite of infected mosquitoes or from a variety of animal-associated activities. Experimental models in lambs have shown that while efficiency of infection per bite is 23%, a single bite is sufficient for infection [40]. While this is generally considered a less common route of exposure in humans, surveillance bias to severe RVFV cases likely underestimates the contribution of vector-borne transmission at the community level. Flooding has been a long-recognized risk factor for human infection, as have dams and irrigation systems which create favorable RVFV habitats [41,42]. Human infections have been traced back to contact with infected animals when they carry out high-risk activities including slaughtering, milking, assisting in animal birth, and handling of abortion materials [33,43]. In addition to household-level exposures, veterinarians and those working in animal husbandry and slaughtering have an increased risk. As with many viruses, this is likely due to droplet exposure or direct inoculation to mucosal membranes, though aerosol transmission has been shown to be associated with encephalitic manifestations of disease in animal models [44]. Similarly, given strong associations between milk consumption and RVFV exposure, consumption of raw milk, blood, and meat may provide additional opportunities for direct exposure via mucous membranes, but is unlikely to result in alimentary disease [45,46]. While transmission is frequently reported in rural regions associated with livestock rearing, urban transmission has recently been described in Kenya, extending potential populations at risk [47]. Direct human-to-human transmission has not been reported.

### Distribution and endemic/epidemic and hyperendemic dynamics

RVFV is widely distributed across the African continent and evidence of infection is present in nearly every country to varying degrees [5,33], and epidemics have been recorded in Saudi Arabia and Yemen. New geographic records of RVFV are not always associated with apparent clinical outbreaks as seems to be the case with recent evidence of human exposure in Türkiye, though these antibody tests have not been confirmed with gold-standard PRNT testing and cross-reactivity remains hypothetical [16]. In fact, transmission patterns and disease incidence have been shown to vary significantly between regions with different vector, environmental, livestock systems, and socio-cultural contexts.

Historically, in epidemic-prone regions including East Africa, RVFV epidemics occurred every 5–10 years; however, smaller and more sporadic RVFV case clusters are now detected with increasing frequency [33]. It remains unclear to what degree these changes are due to changing epidemiologic patterns versus improved diagnosis and surveillance [33]. Traditional models of RVFV epidemiology defined endemic/epidemic patterns in which low levels of interepidemic transmission and transovarial transmission maintained RVFV until climatic conditions led to large epidemics associated with flooding events. It is becoming increasingly clear that RVFV circulates in regions with no reported epidemics, often with higher year-to-year incidence suggesting seasonally fluctuating endemic transmission consistent with vector-borne disease rather than classic endemic/epidemic transmission [38]. Differences in annual vector populations, climate seasonality, rainfall patterns, land use, and sociocultural conditions influence a region’s position in this endemic–epidemic continuum.

Major foci of infection occur in all regions of Africa with varying degrees of spillover risk [5,48,49]. In East Africa, the largest epidemics have primarily been in Kenya, Tanzania, and Somalia, although a new hyperendemic focus of infection has more recently emerged in southwestern Uganda [38,50]. Large epidemics in Kenya such as in 1997–1998 and 2006–2007 are typical of endemic/epidemic dynamics, but since 2008, 91% of cases have been identified in smaller dispersed clusters involving small numbers of livestock rather than large-scale epidemics [51]. Classical East African RVFV epidemics such as that in 2006/2007 have been recognized as sources of transmission to new regions in the Indian Ocean and Arabian Peninsula. In South Africa, both endemic/epidemic dynamics are noted in Western Free State and hyperendemic dynamics noted in KwaZulu-Natal [38,48]. In West Africa, epidemics are centered around the Senegal River basin affecting Senegal and Mauritania, including a recent 2025 epidemic [49,52]. Recurrent epidemics in Madagascar are somewhat unique in that interepidemic transmission does not seem sufficient to maintain viral circulation, and maintenance of transmission appears to be the result of livestock movements resulting in hyperendemicity [12].

Key gaps, highlighted by numerous prior reviews, remain in the understanding of RVFV epidemiology that limit our ability to plan for and test countermeasures. Importantly, these gaps include accurate regional and subregional epidemiologic patterns, as sampling bias from epidemic-prone regions provides an incomplete description of RVFV clinical syndromes and transmission [5,33,53,54]. More broadly, deeper understanding of vector-specific risk and vector ecology across space and land use areas is needed to assess how risk varies spatially. Surveillance systems and outbreak investigations must expand and fully operationalize One Health approaches to simultaneously assess human, livestock, and vector epidemiology. Delineating human risk factors and vaccine uptake approaches in endemic settings will be particularly important to develop effective vaccination strategies within the coming decades.

## Pathophysiology

### Pathology and tropism

Pathologic descriptions of RVF are sourced from a limited number of human infections and are more extensively derived from livestock and animal model data. Following infection of local cells after a mosquito bite or mucosal exposure, dendritic cells and macrophages are infected, resulting in spread to the lymphatic system [55,56]. Upon dissemination in the host, RVFV displays marked hepatotropism, with the liver being the primary site of replication, which can result in extensive hepatocyte necrosis and apoptosis alongside inflammation, hemorrhage, and viral replication in hepatocytes and Kupffer cells [57–59]. Disease is thought to be mediated by a combination of direct viral-mediated damage and dysregulated immune response. Autopsy series have described splenic hemorrhage, atrophy and degeneration of lymphoid follicles, pulmonary congestion and hemorrhage, diffuse gastrointestinal hemorrhages, and renal tubular degeneration [57,58]. RVFV encephalitis is also reported with perivascular cuffing and focal regions of parenchymal necrosis with lymphocytic and mononuclear infiltrate and neuronal infection [44,56,57]. As with many encephalitic viruses, invasion of the central nervous system (CNS) may be mediated via the olfactory nerve or direct infection of blood-brain barrier cells [60,61]. RVFV retinitis and uveitis in a rat model is characterized by upregulation of proinflammatory cytokines, leukocyte invasion, and infection of the optic nerve, uvea/choroid, ciliary body, and ganglion layer of the retina [62]. Another study in humans demonstrated trends for increased levels of anti-retinal antibodies among RVFV retinitis survivors suggesting possible immune-mediated mechanisms [63]. RVFV replicates in chorionic villi of human placenta and trophoblasts in livestock infections leading to spontaneous abortions [56,64].

### RVFV non-structural proteins and immune responses

Control of RVFV infection requires type I interferon (IFN) responses, early induction of inflammation, and timely development of cellular and humoral immune responses. Multiple IFN-deficient animal models develop severe disease, usually manifesting as severe hepatitis [65,66]. NSs is the major virulence factor of RVFV which functions as an IFN antagonist, suppresses host-cell mRNA, and promotes viral translation [67,68]. NSs globally inhibits transcription resulting in decreased IFN and IFN-stimulated gene expression via interaction/degradation of the host transcription factor TFIIH [67]. NSs additionally interacts with PKR leading to ubiquitination and degradation, thereby facilitating viral protein translation while simultaneously suppressing host-cell translation via interactions with PABP1 [68–70]. NSm has also been shown to suppress apoptosis in infected cells [71].

Early induction of inflammatory cytokines and chemokines is critical for protection from lethal infection [72]. However, dysregulated inflammatory responses in tissue are also associated with immune-mediated pathogenesis [73]. Adaptive immune responses lead to likely livelong immunity in humans. The dominant antibodies conferring protection target Gn/Gc and are a correlate of immunity with passive immunization using neutralizing antibodies fully protecting against lethal RVFV infection and vertical transmission [74,75]. Cellular immunity is also critical for clearance of RVFV, particularly from the CNS [65,76].

## Clinical manifestations

RVFV in livestock is characterized by widespread abortions, known as “abortion storms” and mortality in younger animals [77], and this presentation has been leveraged by surveillance systems for the detection of epidemics. In livestock, disease is typically classified as hyperacute, acute, and subacute/inapparent. Hyperacute disease characterized by massive hepatic necrosis is often seen in very young animals such as lambs and reaches approximately 100% mortality [56]. Older juveniles may develop respiratory signs, hemorrhagic nasal discharge, diarrhea, and colic with 10%–60% mortality. Most adult animals present with subclinical or mild disease characterized by fever, weakness, nasal discharge, and interruption of lactation with full recovery. Serologic studies in livestock suggest much more widespread mild/asymptomatic infection in endemic regions. In fact, adult indigenous breed livestock may not have any apparent clinical signs at all which can facilitate silent spread and endemic maintenance [78].

In humans, RVF presents as a wide spectrum from mild subclinical infection to severe manifestations including encephalitis/meningitis, hemorrhagic fever, retinitis, and pregnancy loss with high case fatality rates (CFR) [79]. Similar to livestock studies, seroprevalence studies in humans suggest widespread subclinical presentation, and clinical descriptions of RVF are biased to cases identified via syndromic surveillance during epidemics. Approximately 90%–98% of RVFV infections result in mild to asymptomatic illness with an overall CFR during the 1977 Egyptian epidemic estimated to be 0.3% [7,80]. In Mayotte, modeling studies following an epidemic suggested only 1.5% of infections were identified via active syndromic surveillance and of those 8% developed severe disease resulting in hospitalization [80,81]. Among severe RVF cases CFRs range from 15% to 30% [9,82], and it is generally not well understood why some humans have no apparent disease and some develop severe manifestations.

RVFV has an incubation period of approximately 3–6 days [57]. Mild RVF presents with sudden-onset malaise, fever, large-joint arthralgias, and headache, with some patients reporting diarrhea, emesis, and abdominal pain [7,57,82]. Symptoms typically last one to four days with some patients developing a second febrile period after an initial convalescent period. Severe disease is more strongly associated with exposure to infected animals, possibly due to dose of inoculum or route of infection [83]. Following initial febrile presentation, patients progress to develop a syndrome of jaundice, right upper-quadrant pain, and delirium with or without hemorrhagic/CNS manifestations [84]. Among patients hospitalized in the Saudi Arabian epidemic, 75% experienced hepatic failure, 41% renal failure, 19% hemorrhagic disease, 10% retinitis, and 4% late-onset meningitis/encephalitis with hepatorenal syndrome and disseminated intravascular coagulation (DIC) representing other common complications [10]. Imaging findings of pneumonia, cholecystitis, and pleural effusions have also been noted [15]. Lab findings include anemia, thrombocytopenia, leukopenia, elevated transaminases and bilirubin, elevated creatinine/blood urea nitrogen (BUN), lactate dehydrogenase (LDH), and prolonged prothrombin time (PT)/activated partial thromboplastin time (aPTT) [10,82]. Hemorrhagic disease may manifest as melena, hematemesis, petechial rashes, purpura, and gingival bleeding with CFRs of approximately 50% [7]. Neurologic manifestations include meningismus, photophobia, confusion, lethargy, hallucinations, vertigo, paresis, and cranial nerve palsies [7,57,82]. CNS disease may be delayed 5–60 days following symptom onset [7,57]. Significant ocular disease may include macular, para-macular, or extramacular retinitis 2–7 days following symptom onset with decreased visual acuity [85,86]. Self-resolving anterior uveitis has also been reported in 31% of patients presenting with ocular manifestations [85].

Long-term sequelae to RVF are often characterized by persistent visual deficits, with retinal scarring in 60% of retinitis cases, with only a minority experiencing improvement [85]. A study in Kenya also revealed the presence of visual deficits and retinal damage even in the absence of reported illness among RVFV seropositive individuals [41]. A critical but understudied aspect of RVF in humans is the effect on pregnancy. A recent major study has shown 54% of pregnant women in Sudan with symptomatic RVF experienced spontaneous abortions [87]. However, the effects of mild RVFV infection in pregnancy remain unknown. Additionally, the contribution of RVFV to additional adverse birth outcomes such as low birth weight or preterm birth remains unknown. These critical gaps in knowledge will be critical to clarify to guide future vaccination strategies.

## Diagnosis

Clinical diagnosis of RVF is unreliable due to non-specific and variable presentations. Diagnostic testing relies generally on reverse transcriptase polymerase chain reaction (RT-PCR) or detection of anti-RVFV IgM and is generally only available at reference laboratories [33,88]. Anti-RVFV IgG testing can be used to determine prior exposure and retrospective diagnosis. Viremia lasts approximately 2–8 days, with IgM detectable 3–4 days after infection and remains detectable for 15–20 days, with some reports of detection up to 50 days [89,90]. Most RVFV detections have been made using IgM detection with or without combination RT-PCR testing [33]. Serologic testing is achieved via immunofluorescence assay (IFA) or more commonly enzyme-linked immunosorbent assay (ELISA) with a number of commercial and in-house assays available.

Access to RVFV diagnostics remains limited in under-resourced settings, limiting clinical detection, particularly in the absence of detected epidemics and mobilization of outbreak response teams. Improved access to standardized, validated, and affordable diagnostics will be critical for improved surveillance, case detection, and implementation of vaccines and therapeutics in clinical trial and, eventually, clinical settings. Rapid point-of-care testing including lateral flow assays (LFA) are in development and offer promising opportunities for more widespread rapid detection [88,91]. However, the diagnostic limitations of LFAs will require coordination with confirmatory serologic and/or RT-PCR assays.

## Treatment

### Current treatment

Currently, there are no approved RVFV-specific therapeutics [92]. Current treatment for RVF in humans largely depends on supportive care including renal replacement therapy, mechanical ventilation, blood pressure support, fluid resuscitation, and management of coagulopathy, if indicated [89]. Retinitis has been treated with topical and oral corticosteroids with some reported improvement in a very small retrospective case-control study, with more studies required for confirmation [93].

### Antivirals, host-directed therapies, and monoclonal antibodies

Among virus-directed therapies, several small-molecule antiviral compounds show promise in the treatment of RVFV. Ribavirin has been the most frequently evaluated antiviral *in vitro* and in animal models. While ribavirin does inhibit viral replication *in vitro* and show some efficacy in preventing fatal hepatitis in animal models, survivors may develop delayed-onset encephalitis, potentially due to inability of ribavirin to cross the blood-brain-barrier [94,95]. Additionally, ribavirin was used in the Saudi Arabian epidemic, and while outcomes were not fully published, reports indicate lack of efficacy was observed [89]. Favipiravir shows improved efficacy compared to ribavirin in animal models, but in rat models approximately 10% still develop delayed-onset encephalitis [96]. Other pre-clinical studies have shown varying *in vivo* efficacy using the nucleoside analog galidesivir, the IFN agonist tilorone, the TLR2 ligand Pam_3_CSK_4_, sorafenib, and rapamycin [92,97–99].

Perhaps the most promising therapeutic candidate is the use of monoclonal antibodies. Multiple candidate neutralizing monoclonal antibodies targeting the Gn/Gc complex have been identified which demonstrated 100% survival in a murine model when administered as pre-exposure prophylaxis or 2 days post-infection as well as 40% survival when administered as late as 4 days post-infection [74]. Follow-up studies with differing cocktails of these antibodies have demonstrated protection against encephalitis and vertical transmission in lethal rat models [75,100]. A cocktail of two monoclonal antibodies has now progressed through phase I clinical trials and may offer opportunities for therapeutic interventions in future epidemics. However, as with other countermeasures, therapeutic clinical trial endpoints will be difficult to achieve because most identified infections will likely be mild without development of severe disease. Additionally, while monoclonal antibodies may offer rapid protection with mixed prophylactic/therapeutic potential, their cost, deployment, and implementation may be challenging in low-resource settings. While late-onset encephalitis has not been observed in animal models of RVFV following monoclonal antibody treatment, careful monitoring for this outcome or recrudescence should be considered during human clinical trials given the theoretical risks and similar observations following monoclonal antibody treatment of Ebola virus disease [101,102].

## Prevention

### One Health approaches

Due to the critical role of livestock in RVFV ecology and epidemiology, integrated One Health approaches involving medical, veterinary, agricultural, and environmental agencies are critical in responding to RVFV epidemics. Too often, sporadic human case detections lead to investigations which reveal more extensive evidence for undetected human and livestock transmission [103]. To date, few studies truly incorporate One Health designs and collaboration in interepidemic settings [33]. Future strategies should incorporate more active RVFV surveillance and identification of overlapping risk factors in human-animal-environmental frameworks.

Successful One Health prevention strategies have incorporated livestock vaccination and climate-based RVFV epidemic forecasting. Livestock are thought to represent the bulk of mammalian infections and livestock vaccination is thought to have larger impacts on RVFV community transmission than human-based vaccination strategies. Widely used vaccines include the live-attenuated Smithburn strain (among other attenuated strains), although these may have residual virulence and teratogenic effects in pregnant animals [104,105]. Newer attenuated vaccines such as Clone 13 (C-13) have been developed and licensed, but availability remains low [104]. Even when vaccines are available, uptake outside of epidemic periods remains low and reactive epidemic vaccination campaigns have reported only limited success.

RVFV forecasting is possible in some settings given associations with weather patterns, in particular El Niño-associated flooding in East Africa and La Niña in South Africa. A satellite and climate-based model developed for use in East Africa has accurately predicted epidemics with 2–6 week lead time, enabling reactive strategies to be implemented [106,107]. Similar models have also been developed for South Africa with more variable accuracy. However, these models sometimes overestimate outbreak scale and underestimate risk of smaller localized clusters of transmission [108,109]. Additionally, these models do not account for sociocultural factors including livestock immunity and transport. Despite these limitations, climate-based models remain important tools for predicting spatial risk for large classic flood-associated RVFV epidemics.

In response to a more contemporary understanding of RVFV epidemiology in places such as East Africa, where outbreaks are increasingly much smaller, sporadic, and decoupled from weather patterns, there is a need to develop predictive models that incorporate metrics important in endemic disease maintenance and outbreak potential. One hypothesis for the lack of large outbreaks is higher and more consistent baseline exposure risks that result in an inadequate pool of susceptible hosts to generate a large, and thus, detectable outbreak. Systems dynamics models that track immunity levels and can manage the integrated risk with weather patterns could improve forecasting tools. For predicting and informing risk associated with spread of localized case clusters, models that integrate fine-scale livestock movements are likely to prove useful and can be leveraged to assess the theoretical efficacy of control programs [110].

### RVFV vaccines

**Fig 3:**
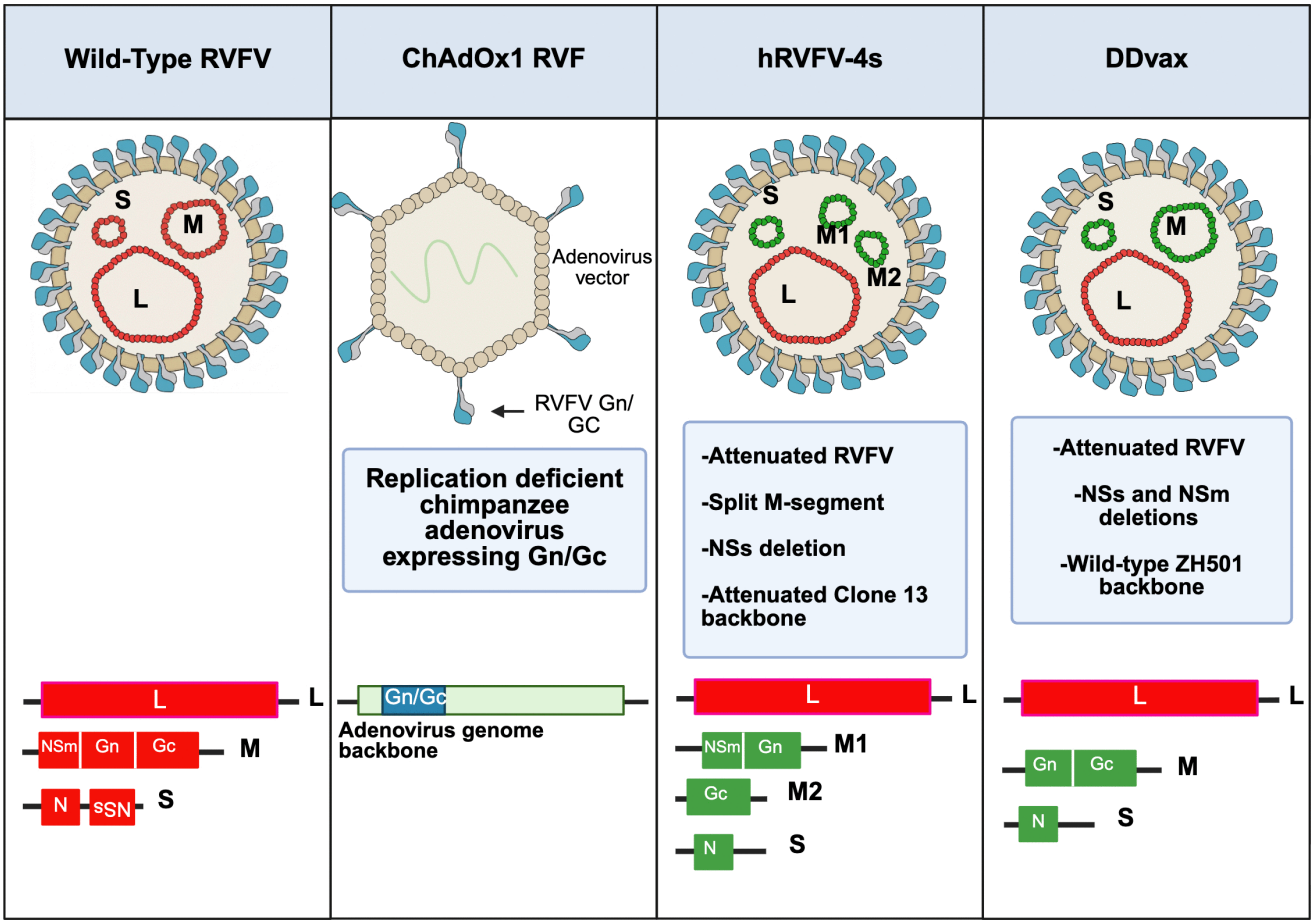
Advanced Rift Valley fever virus vaccine candidates. This diagram presents the leading CEPI-supported RVFV vaccine candidates currently or planned to undergo clinical trials. Wild-type RVFV structure and genome organization are displayed for comparison to live-attenuated vaccine strains. The ChAdOx1 RVF candidate is a viral-vectored vaccine using a replication-deficient chimpanzee adenovirus vector expressing RVFV Gn/Gc and is currently undergoing phase 2 clinical trials. hRVFV-4s is a live-attenuated vaccine in which transcriptional and packaging efficiency is attenuated by splitting the M genome segment alongside deletion of the virulence factor NSs and has completed a phase 1 clinical trial. DDvax is a live-attenuated vaccine in which NSm and NSs are deleted, pending planned phase 1/2 clinical trials. Created in BioRender. Dawes, B. (2026) https://BioRender.com/wifvwvc.

There are several RVFV vaccine candidates in advanced development following its designation by WHO and the Coalition for Epidemic Preparedness Innovations (CEPI) as a priority pathogen (Fig 3) [105]. While there are currently no licensed vaccines for human use, historic vaccinations have been used including a formalin-inactivated vaccine (TSI-GSD-200) which has been used in military personnel or lab workers but required at least four doses [111]. A live attenuated vaccine strain, MP-12, was developed under serial passage in the presence of a mutagen and completed phase 1 and 2 clinical trials with protective neutralizing antibody titers maintained for at least one year [112].

In recent decades, a large number of next-generation vaccine candidates have been developed and evaluated in pre-clinical models [113,114]. Of these candidates, there are three advanced candidates currently or planned to enter clinical trials. A chimpanzee adenovirus-vectored vaccine candidate (ChAdOx1 RVF) developed at Oxford University and administered as a single dose has completed phase 1 clinical trials with strong neutralizing antibody and T-cell responses [115]. ChAdOx1 RVF is now undergoing a phase 2 clinical trial in Kenya. The hRVFV-4s live-attenuated virus vaccine candidate is a recombinant RVFV in which NSs is deleted, and the M segment is divided into 2 separate segments resulting in an avirulent phenotype [116]. hRVFV-4s has also completed a phase 1 clinical trial demonstrating favorable immunogenicity [117]. A second live-attenuated vaccine, DDVax consists of RVFV containing deletions to both NSs and NSm leading to a highly attenuated phenotype which has demonstrated efficacy in multiple animal models including non-human primates with plans to advance to clinical trials in the coming years [118,119]. Most recently, CEPI has invested in the development of a RVFV mRNA vaccine developed by Afrigen in South Africa, although this potential candidate remains in development with no data yet available.

## Future priorities

The coming decades offer a chance to revolutionize the understanding, treatment, and control of RVFV if coordinated One Health approaches are implemented. Expanding awareness and diagnostic access is reshaping the understanding of RVFV epidemiology by uncovering regions of hyperendemic transmission and expanding the range in which RVFV has been detected. Deeper understandings of regional ecologies and transmission dynamics between vectors, livestock, wildlife, and humans will allow for more targeted RVFV surveillance and control strategies. However, this will require widespread coordinated epidemiologic and surveillance studies leveraging human, livestock, vector, and environmental monitoring systems. Ultimately, better understandings of RVFV dynamics could allow for more accurate epidemic forecasting models to be developed. With regards to human disease, further studies are needed to clarify routes of transmission and risk factors in local sociocultural contexts which can lead to more effective public health interventions.

The advent of potential therapeutics in the form of monoclonal antibodies and antiviral compounds as well as vaccines in clinical evaluation are promising developments which may rapidly transform the RVFV clinical landscape nearly 100 years after its discovery. However, given the unpredictability of epidemics and sporadic detection of interepidemic cases, these clinical trials will be technically challenging and large-scale phase 3 trials may be difficult to complete. In the event of vaccine approval, use cases will need to be defined. It is likely that these vaccines will be developed for stockpiles to be deployed in the event of large epidemics. However, there must also be consideration for preemptive vaccination strategies in hyperendemic regions, in epidemic-prone regions with accurate forecasting systems, and of those with specific occupational risk such as slaughterhouse workers, farmers, and veterinarians. Coordinated One Health vaccination strategies will be vital to limiting the vast economic, human and animal health impacts of outbreaks. A particularly alarming unanswered issue is the burden of adverse pregnancy outcomes in endemic regions. The burden of maternal-child health outcomes may influence if women of childbearing age or pregnant women will constitute key target populations for future vaccines. Use of live-attenuated vaccines in this population may be more challenging given the potential for teratogenicity observed for some live-attenuated vaccines in animals and will require careful safety review. New and emerging countermeasures offer promising opportunities to improve the control this globally important pathogen. However, their development and implementation must be grounded in the needs, perspectives, and intended use contexts of populations most affected by RVF. Countermeasures developed without meaningful consideration of both endemic and epidemic-prone regions risk limited feasibility, challenges in demonstrating efficacy in trial settings, and the continued reinforcement of global health inequities that hinder sustained global control of RVFV transmission.
